# Nandrolone induces a stem cell-like phenotype in human hepatocarcinoma-derived cell line inhibiting mitochondrial respiratory activity

**DOI:** 10.1038/s41598-020-58871-1

**Published:** 2020-02-10

**Authors:** Francesca Agriesti, Tiziana Tataranni, Consiglia Pacelli, Rosella Scrima, Ilaria Laurenzana, Vitalba Ruggieri, Olga Cela, Carmela Mazzoccoli, Monica Salerno, Francesco Sessa, Gabriele Sani, Cristoforo Pomara, Nazzareno Capitanio, Claudia Piccoli

**Affiliations:** 1 Laboratory of Pre-Clinical and Translational Research, IRCCS-CROB, Referral Cancer Center of Basilicata, 85028 Rionero in Vulture, Italy; 2 Department of Clinical and Experimental Medicine, University of Foggia, via L. Pinto c/o OO.RR., 71100 Foggia, Italy; 3 Department of Medical, Surgical Sciences and Advanced Technologies “G.F. Ingrassia”, University of Catania – A.O.U. “Policlinico - V. Emanuele”, via S. Sofia, 87 – Sector 10, Building B – 95123, Catania, Italy; 4 Department of Neuroscience, Section of Psychiatry, Università Cattolica del Sacro Cuore, Roma, Italy; 5 Department of Psychiatry, Fondazione Policlinico Universitario Agostino Gemelli IRCCS, Roma, Italy

**Keywords:** Biochemistry, Cancer, Cell biology, Stem cells

## Abstract

Nandrolone is a testosterone analogue with anabolic properties commonly abused worldwide, recently utilized also as therapeutic agent in chronic diseases, cancer included. Here we investigated the impact of nandrolone on the metabolic phenotype in HepG2 cell line. The results attained show that pharmacological dosage of nandrolone, slowing cell growth, repressed mitochondrial respiration, inhibited the respiratory chain complexes I and III and enhanced mitochondrial reactive oxygen species (ROS) production. Intriguingly, nandrolone caused a significant increase of stemness-markers in both 2D and 3D cultures, which resulted to be CxIII-ROS dependent. Notably, nandrolone negatively affected differentiation both in healthy hematopoietic and mesenchymal stem cells. Finally, nandrolone administration in mice confirmed the up-regulation of stemness-markers in liver, spleen and kidney. Our observations show, for the first time, that chronic administration of nandrolone, favoring maintenance of stem cells in different tissues would represent a precondition that, in addition to multiple hits, might enhance risk of carcinogenesis raising warnings about its abuse and therapeutic utilization.

## Introduction

Nandrolone (ND), a synthetic testosterone analogue, is one of the most commonly abused anabolic androgenic steroids (AAS) worldwide. For its improved anabolic properties ND is widely clinically applied in treatment of chronic diseases associated with catabolic state such as burns, cornea healing and osteoporosis^1^. Recently, positive effects of nandrolone have been also described in cancer, in which ND seems to have several therapeutic applications. For example, the greater ratio of myotrophic:androgenic properties, resulting in appetite stimulation and increased red blood cell production, makes it suitable to fight neoplastic cachexia and anemia associated with leukemia^1^. In addition, its ability to impair the expression of key enzymes for the steroids biosynthesis such as CYP17A1, produces anti-proliferative effects^2^ similarly to that of inhibitors of androgen receptor (AR), in prostate and breast cancers^3,4^. However, despite the clinical beneficial effects of ND^5^, there are no clear guidelines regarding the optimal therapeutic doses of androgens or long-term safety data.

Unfortunately, ND and AAS are also used for illicit self-administration to improve athletic physical performances and their abuse is associated with serious adverse effects^6^. The most relevant side-effect is hepatotoxicity since human liver expresses estrogen and androgen receptors and experimentally both androgens and estrogens have been implicated in stimulating hepatocyte proliferation, probably causing liver tumor^7,8^. Accordingly, several case reports highlight a link between AAS abuse and risk of developing the male-dominant hepatocellular carcinoma (HCC)^9–12^. Several studies supported the androgen/AR axis as a pivotal factor that contributes to gender difference in HCC etiology^13–16^. Furthermore, androgen signaling is the critical determinant in male gender development, suggesting its prominent role in the regulation of normal or cancer stem/progenitor cells (CS/PCs)^17^. CS/PCs are a small subgroup of cancer cells, also defined as stem cell-like, with high self-renewal, extensive proliferation and strong tumorigenesis capacity, playing an established role in oncogenesis of various cancers, including HCC^18,19^. In agreement with their dominant role in liver regeneration, mature hepatocytes are emerging as the cell of origin of HCC. However, the relationship between the cancer cell of origin (CCO) and hepatic cancer stem cell (HCSC) is unknown^20^. A recent study revealed the relationship between androgens and HCSC maintenance demonstrating that androgens and AR participated in cancer stem cells (CSCs) regulation through the *nanog* related pathway, a potent positive regulator of HCC stemness^21^.

Emerging evidence shows that the metabolic phenotype of cancer cells facilitates their plasticity and may be specifically associated with metastasis and therapy-resistance. Moreover, cancer cells can switch their metabolism phenotypes in response to external stimuli for better survival. Likewise, many recent studies have implicated metabolic mechanisms as major regulators of pluripotent stem cells properties and mitochondrial functions as controller of stem cell maintenance/differentiation in several cell types^22–27^. To the best of our knowledge, no studies about androgens and ND influence on mitochondrial bioenergetic function in cancer cells have been reported so far. Therefore, the aim of this study was to investigate the effect of nandrolone on proliferation and differentiation of HCC cells examining the interplay between modulation of mitochondrial oxidative metabolism and ND ability to drive metabolic plasticity of normal/cancer stem cell differentiation and cellular reprogramming.

## Results

### Nandrolone suppresses HepG2 cells proliferation

**Figure 1:**
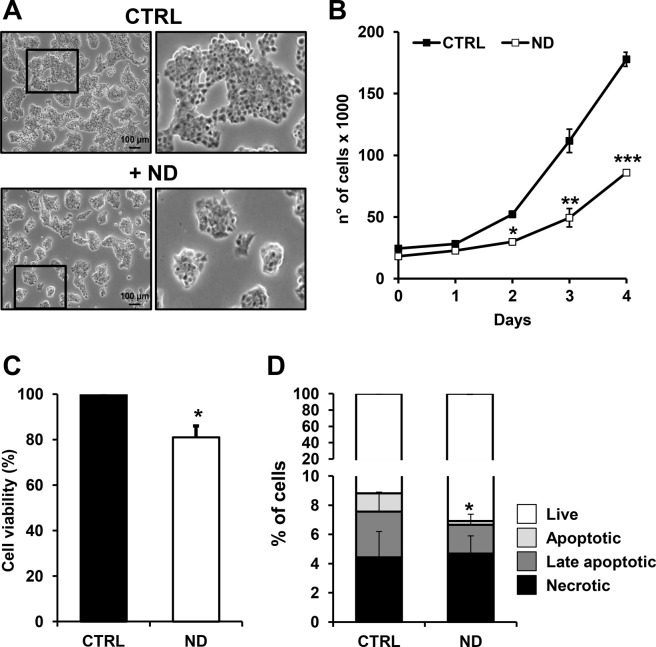
Effect of nandrolone on cell viability. (**A**) Phase-contrast images of cultured HepG2 cells in ± 80 μM nandrolone for 72 h. Scale bars, 100 μm. The shown optical micro-photographs on the left are representative of several independent biological replicates yielding similar results; digital magnifications of selected areas are also shown on the right panel. (**B**) Cell growth curves of HepG2 seeded at the same density in presence or absence of nandrolone and counted every 24 h at the indicated times; the values shown are means of three independent ± SEM time-courses for each condition (where not visible the error bar is within the size of the symbol); ^*^P < 0.05, ^**^P < 0.01, ^***^P < 0.005 vs relative CTRLs. (**C**) Effect of nandrolone on cell viability assessed by MTS assay, as described in Materials and Methods. Cell viability is expressed as the percentage (%) of untreated cells. The data shown are means ± SEM of at least three independent experiments; ^*^P < 0.05. (**D**) Apoptosis measurement of untreated and ND**-**treated cells. HepG2 cells were incubated with annexin V and propidium iodine (PI) and the internal-sample percentage of early apoptotic, late apoptotic and necrotic cells assessed by flow cytometry as detailed in Materials and Methods. The superimposed bar graph shows the average values ± SEM of three independent experiments; ^*^P < 0.05. CTRL: ethanol-treated Control cells, ND: nandrolone-treated cells.

In order to test the effect of nandrolone on cell proliferation, HepG2 cells were treated with the drug at concentrations ranging from 2.5 to 160 μM for 7 days (data not shown). Based on the results attained, a treatment of 80 μM of nandrolone for 72 h was chosen since it caused a marked inhibition of the cell growth still preserving cell viability. Figure 1A shows the phase contrast imaging of nandrolone-treated cells, displaying formation of smaller cell clusters. The growth curve analysis showed a significant inhibitory effect of nandrolone already evident after 48 h treatment (Fig. 1B). However, cell viability, assessed by MTS assay, resulted to be only slightly affected (Fig. 1C). Accordingly, following nandrolone treatment the relative amount of necrotic HepG2 cells, measured by the annexin V/PI assay, did not change significantly with early and late apoptotic cells resulting even decreased (Fig. 1D).

**Figure 2:**
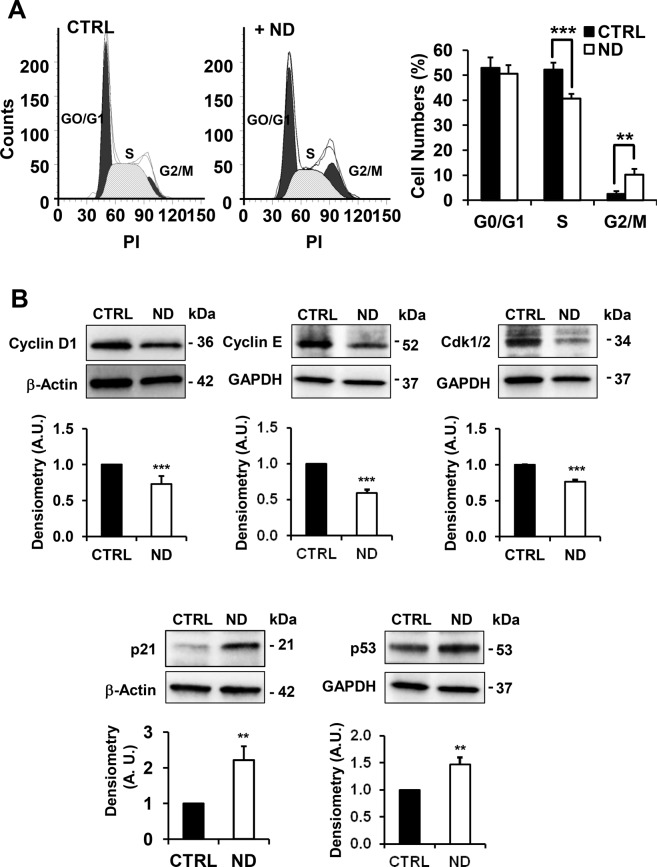
Effect of nandrolone on cell cycle. (**A**) Cell cycle analysis of HepG2 nandrolone treated cells (ND) using ModFit software (Verity Software House). Histogram plots were shown on the right. Data, expressed as percentage of total events analyzed, are the means ± SD of three independent experiments; ^**^P < 0.01; ^***^P < 0.005. PI: propidium iodide. (**B**) Protein expression levels of key regulators of cell cycle progression, Cyclin D1, Cyclin E, Cdk1/2, p21 and p53, assayed by Western blotting in untreated and 80 μM nandrolone-treated cells for 72 h. Upper panel: a representative cropped immunoblot. Full-length blots are presented in Supplementary Fig. S1. Bottom bar histogram: densitometry analysis normalized to β-actin as means ± SEM of three independent experiment; ^**^P < 0.01; ^***^P < 0.001. A.U.: arbitrary unit.

Cell cycle assay performed by flow cytometry showed in ND-treated cells a lower percentage of cells in S phase associated with a higher percentage in G2 phase as compared with untreated cells, thus indicating a G_2_/M cell cycle arrest (Fig. 2A). Finally, the expression of key proteins involved in the regulation of cell cycle progression, determined by western blot analysis, revealed in ND-treated cells downregulation of Cyclin D1, Cyclin E and Cdk1/2 required for progression through the G1 phase of the cell cycle and, conversely, a significant up-regulation of p21, a known cyclin-dependent kinase inhibitor and of p53, an activator of p21 (Fig. 2B). All these observations suggested that ND exerted a cytostatic rather than a cytotoxic effect.

### Nandrolone negatively affects respiratory chain efficiency and induces mitochondrial ROS production

**Figure 3:**
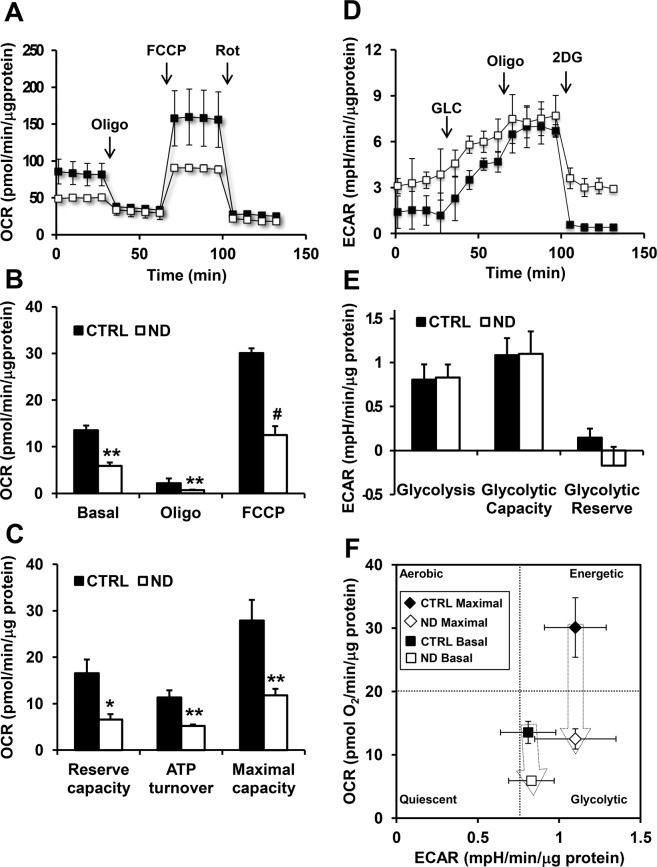
Metabolic flux analysis of Nandrolone-treated HepG2 cells. Representative oxygen consumption rates (OCR) (**A**) and extra cellular acidification rates (ECAR) (**D**) profile of HepG2 cells treated with vehicle (CTRL) or 80 μM nandrolone (ND), assayed by the Seahorse XFe96 Analyzer as described under Materials and Methods. Oligomycin (Oligo), FCCP, and Rotenone (Rot) were added at the indicated points in **A**. Glucose (GLC), Oligo and 2-deoxyglucose (2DG) were added at the indicated points in **D**. The histograms in **B**,**E**, show OCR and ECAR normalized to the protein content of the cells removed from each well at the end of the assay. OCR in **B** Basal, resting OCR; Oligo, OCR measured after the addition of the ATP synthase inhibitor oligomycin also referred as proton leak; FCCP, OCR measured after the addition of the uncoupler FCCP eliciting the maximal respiratory capacity. The OCR were corrected for the residual OCR measured after the addition of the CxI inhibitor rotenone (not shown). ECAR in **E** Glycolysis, resting ECAR, measured after the addition of glucose and corrected for the 2DG-insensitive ECAR; Glycolytic Capacity, ECAR measured after the addition of oligomycin and refers to the maximal glycolytic activity with the OxPhos inhibited; Glycolytic Reserve, difference between ECAR measured in the presence of oligomycin and under resting conditions. (**C)** The histograms show Reserve Capacity, ATP turnover and maximal capacity computed as described in Materials and Methods. (**F**) Energy map obtained plotting the basal and maximal OCR and basal and maximal ECAR measured in **A**,**D** The values are means ± SEM of three independent experiment carried out in 3 technical replicates under each condition; ^*^P < 0.05; ^**^P < 0.01; ^#^P < 0.001.

Since cell proliferation requires a larger bioenergetic support we wondered whether nandrolone affected cell metabolism by impairing mitochondrial oxidative phosphorylation (OxPhos). To this aim, by using the Seahorse extracellular flux analyzer, we simultaneously measured the mitochondrial oxygen consumption rate (OCR) and the extracellular acidification rate (ECAR). As shown in Fig. 3A–C treatment of HepG2 cells with ND resulted in a significant 50% inhibition of the OCR under basal condition as compared with untreated cells. A similar extent of inhibition was observed in the presence of the FoF1 ATP-synthase inhibitor oligomycin and of the uncoupler FCCP. Consequently, both the reserve capacity and the OCR linked to the ATP synthesis/turnover were significantly inhibited. Conversely, no significant changes of the ECAR, which provides an indirect measure of the glycolytic flux, were observed under all the condition tested (Fig. 3D,E). Independent measurements of the cellular lactate confirmed the SeaHorse ECAR-data (Supplemental Fig. S2). Correlation of the OCRs and ECARs in the energy map clearly showed that ND caused dampening of the bioenergetic capacity in HepG2 cells with a major effect on the mitochondrial respiratory chain and consequently of the OxPhos system consistent with the inhibition of the cell cycle and of the cell growth (Fig. 3F).

**Figure 4:**
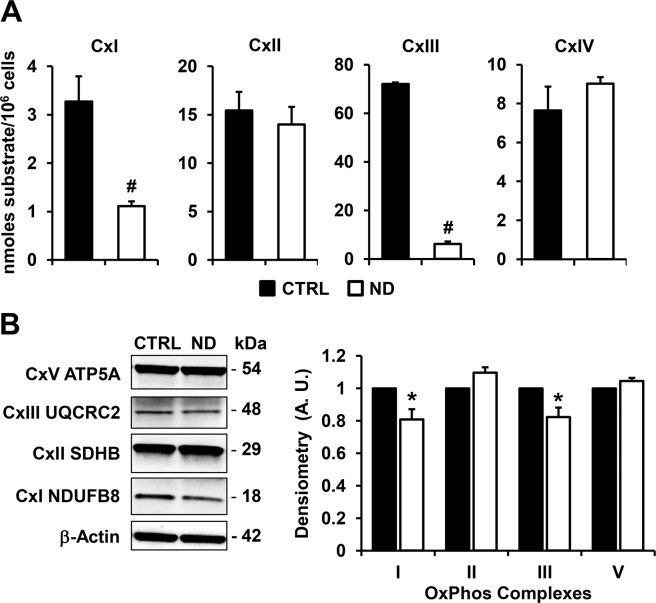
Effect of nandrolone on the mitochondrial respiratory chain complexes. (**A)** Activity of mitochondrial respiratory chain complexes. Cells were lysed and assayed by spectrophotometric assays under condition of saturating substrates as detailed in Materials and Methods. CxI, NADH dehydrogenase; CxII, Succinate dehydrogenase; CxIII, Coenzyme Q-cytochrome c oxidoreductase; CxIV Cytochrome c oxidase. The results are expressed as nmoles of substrate transformed/min/10^6^ cells and the bars indicate the means ± SEM of 4 independent biological replicates for each conditions; ^#^P < 0.001. (**B)** Expression of the OxPhos complexes. Left panel: representative cropped immunoblot of the OxPhos complexes protein expression in untreated (CTRL) and Nandrolone-stimulated (ND) cells using a cocktail of specific antibodies as detailed in Materials and Methods, full-length blots are presented in Supplementary Fig. S3**;** β-actin was used as loading control. Graph bars on the right show the average (±SEM) of data resulting from densitometric analysis of three independent blots; ^*^P < 0.01.

To support the above observation the specific activity of the respiratory chain complexes (CxI, CxII, CxIII, CxIV) was assessed by spectrophotometric assays. The results attained clearly showed for ND-treated cells a marked inhibition of CxI (NADH-dehydrogenase) and, even more consistent, of CxIII (cytochrome c reductase) while no changes were observed for the activities of both CxII (succinate dehydrogenase) and CxIV (cytochrome c oxidase) (Fig. 4A). Despite the strong reduction in the activity of CxI and CxIII, their protein expression levels evaluated by western blot, was only slightly affected (no change in the content of CxII and CxIV was observed) (Fig. 4B). This last result ruled out an effect of nandrolone on the respiratory chain complexes expression/biogenesis.

It is worth noting that both CxI and CxIII require coenzyme Q10 as co-substrate for their catalysis. Thus we decided to test the impact of ND on the kinetic parameters of CxIII. To overcome the limitation linked to the availability of the biological sample, we used mitochondria isolated from bovine heart and followed spectrophotometrically the initial rates of cytochrome c reduction elicited by graded concentration of the Q10 analogue decylubiquinol (dUQ2) in the absence or in the presence of different concentration of ND. The results obtained, shown as reciprocal plot in Fig. S4, indicate a competitive modality of inhibition. The Ki of nandrolone estimated by Dixon plot was about 300 μM. This effect might be accounted for the hydrophobic properties shared between ubiquinol and nandrolone.

**Figure 5:**
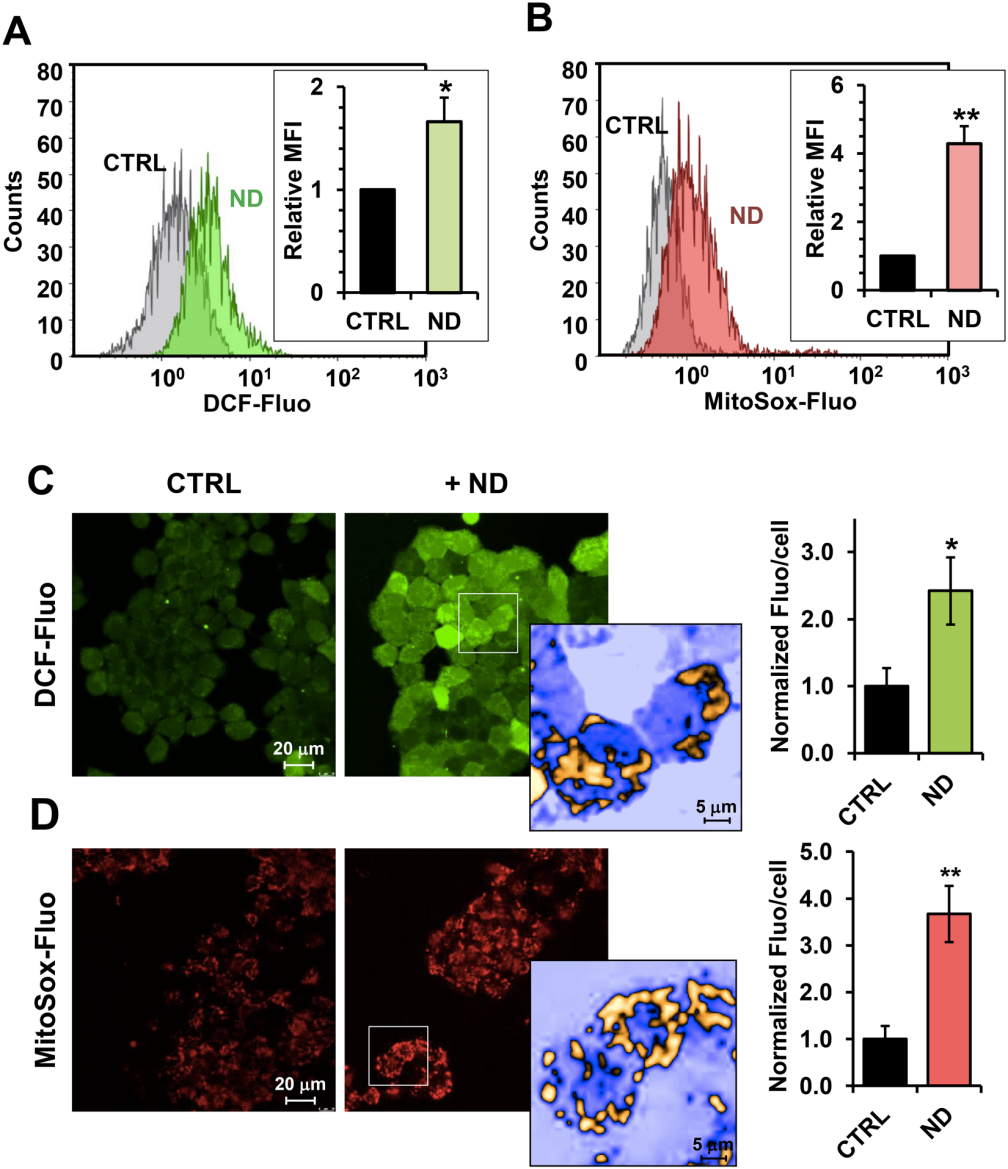
Nandrolone treatment results in unbalance of the cellular redox state of HepG2 cells. Nandrolone elevates ROS production in HepG2 cells. (**A,B)** Flow-cytometric analysis of ROS production assayed by the fluorescent peroxide probe DCF-DA (**A**) and the mitochondrial superoxide anion probe MitoSox (**B**) in untreated (grey areas) and Nandrolone-stimulated (colored areas) cells. Panel on the left: representative flow-cytometric histogram plots. The bar graph in the insets shows the mean intensity of the DCF-DA and MitoSox-related fluorescence (MFI) expressed as fold-change of the untreated cells and are means ± SEM of three independent experiments. ^*^P < 0.05; ^**^P < 0.01. (**C**,**D)** Representative LS**C**M imaging of ROS production in living HepG2 cells treated with nandrolone and assessed by DCF-DA (**C**) and MitoSox (**D**) probes respectively. An enlarged detail of the optical field (square) and rendering of nandrolone-treated cells is shown on the right of each panel (and rendered in false colors). The images are representative of three different preparations yielding similar results. The histograms on the right of panels **C** and **D** show quantification of the fluorescence/cell elaborated by the freeware software ImageJ (https://imagej.nih.gov/ij/); the values are means ± SEM of at least ten different optical fields, each containing 30–50 cells, from 3 independent experiments; ^*^P < 0.05; ^**^P < 0.01.

Inhibition of CxI or CxIII is often associated with production of reactive oxygen species (ROS)^28^. Therefore, we measured ROS production by using the fluorescent probes dichlorofluorescein-diacetate (DCF-DA) and MitoSox, which detect intracellular superoxide and intramitochondrial superoxide anion respectively. Flow cytometry analysis showed higher levels of ROS in nandrolone-treated sample compared to the control assayed by both DCF-DA and MitoSox (Fig. 5A,B). This observation was confirmed by confocal microscopy imaging revealing a significant increase of the fluorescent signal in ND-treated cells regardless of the probe used (Fig. 5C,D). To note, the DCF-fluorescence displayed a more intense signal in fragmented intracellular compartment alike the Mitosox-related fluorescence. These data clearly suggest that nandrolone induces a pro-oxidative state in HepG2 with mitochondria being the main source of ROS.

### Nandrolone triggers the expression of stemness markers in different *in vitro* models

**Figure 6:**
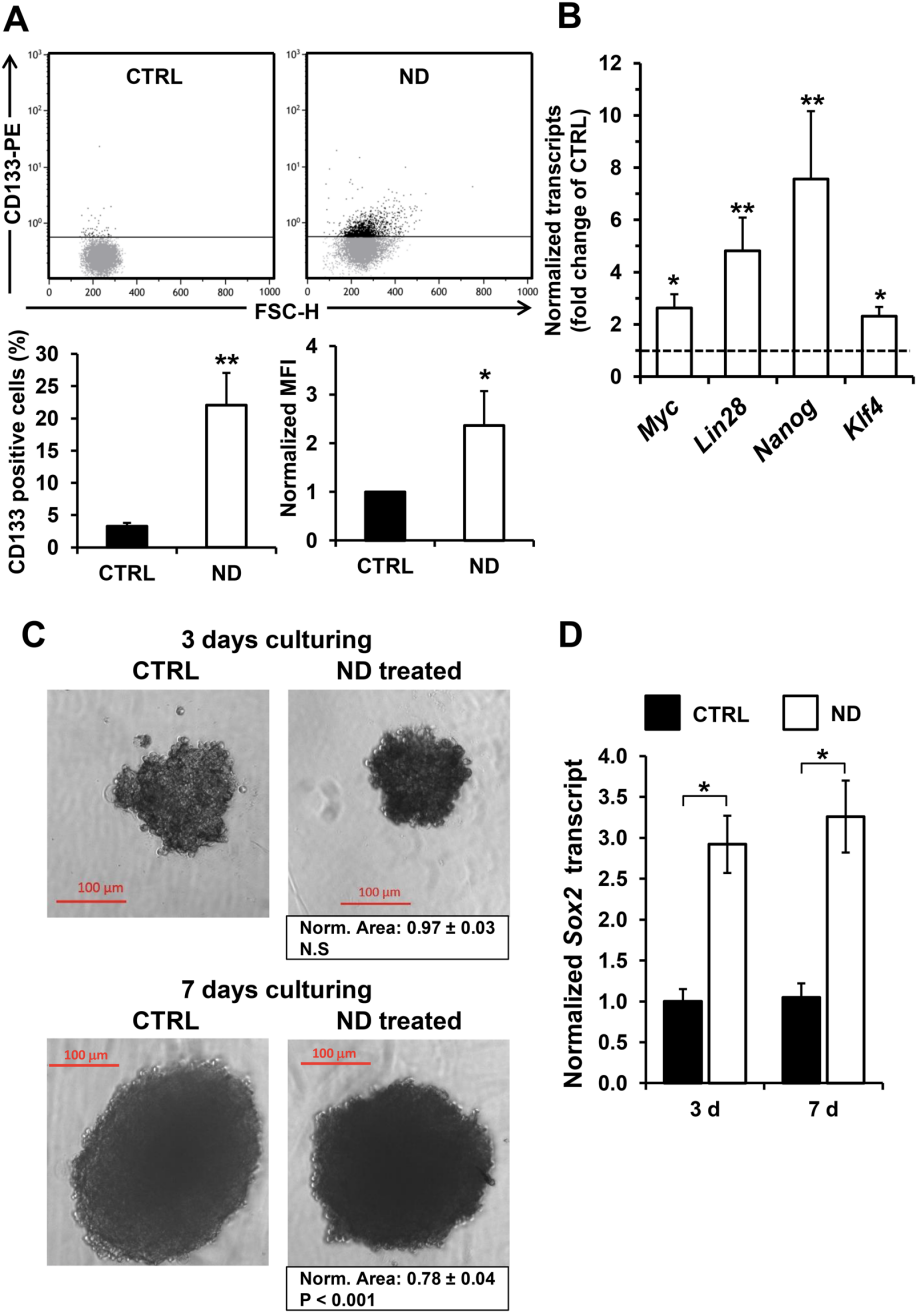
Nandrolone causes a shift toward an immature state (stem cell-like phenotype) in hepatoma cells. (**A)** Flow- cytometric analysis of cancer stem cell surface marker CD133 after 80 μM nandrolone treatment. Representative dot-plots of untreated (CTRL) and nandrolone-treated (ND) cells; Graph bars on the bottom show the average (±SEM) of data resulting from quantification of percentage of CD133 positive cells and from normalized mean fluorescence intensity (MFI) of six independent biological experiments; ^*^P < 0.05; ^**^P < 0.01. FSC-H: forward scatter height. (**B)** Expression of stemness transcription factors resulted up-regulated after nandrolone treatment. Quantitative real‐time‐polymerase chain reaction analysis of transcripts for *Myc, Lin28, Nanog*, and *Kfl4* in HepG2 treated cells is shown as histograms. The values are means ± standard error of the mean (SEM) of normalized transcript levels of six independent biological experiments, ^*^P < 0.05; ^**^P < 0.01; ^#^P < 0.001 versus untreated. (**C)** Effect of nandrolone on HepG2-derived spheroid. Cells were grown for 3 and 7 days under condition of 3D culturing (see Materials and Methods) without (CTRL) and with 80 μM nandrolone (ND treated). The micrographs are representative of two independent experiments; below the “ND treated” panels the averaged spheroid area normalized to the respective CTRL is indicated. (**D)** qRT-PCR analysis of the *Sox2* transcript level in untreated and nandrolone-treated HepG2-derived spheroids, normalized to CTRL sample at the lower timepoint (i.e. 3 days). Experimental conditions as in panel C. Bars are averaged normalized values ± SEM of three independent preparations; ^*^P < 0.05.

CD133 is a surface cell marker identifying a subset of cancer cells, including hepatocellular carcinomas, with stemness properties. Figure 6A shows that treatment of HepG2 cell line with nandrolone caused an almost five fold increase of CD133 positive HepG2 cells, assessed by flow-cytometry. Notably, the MFI in ND-treated cells also increased but not of the extent of the percentage of the CD133^+^ cells. This would suggest that ND is mainly effective in inducing the expression of the stemness marker in CD133^−^ cellular subset rather than enhancing its expression in CD133 cells. However, the possibility that nandrolone enhances the stemness marker in very-low-CD133 expressing HepG2 cells cannot be ruled out.

Furthermore, analysis of the stemness markers *Myc, Lin28, Nanog*, and *Klf4* by real time PCR showed a significant upregulation in nandrolone-treated cells with a marked effect on *Nanog* and *Lin28* gene expression (Fig. 6B). The impact of nandrolone was also tested on the ability of HepG2 to form spheroids. Figure 6C shows that nandrolone treatment of HepG2 under 3D culturing condition resulted in decrease of the spheroid area which was significant after 7 days culturing. Most notably the expression of *Sox2*, a transcription factor driving cancer stemness^29^, increased significantly already after 3 days of spheroid culturing (Fig. 6D) Housekeeping gene expression was not substantially changed by ND treatment neither in 2D nor in 3D cultures as compared with vehicle-treated samples (Supplementary Fig. S5). These observations would suggest a change in the nandrolone-treated HepG2 cells toward a more stem cell-like phenotype that is consistent with the observed nandrolone-induced more quiescent glycolysis-relying metabolism.

**Figure 7:**
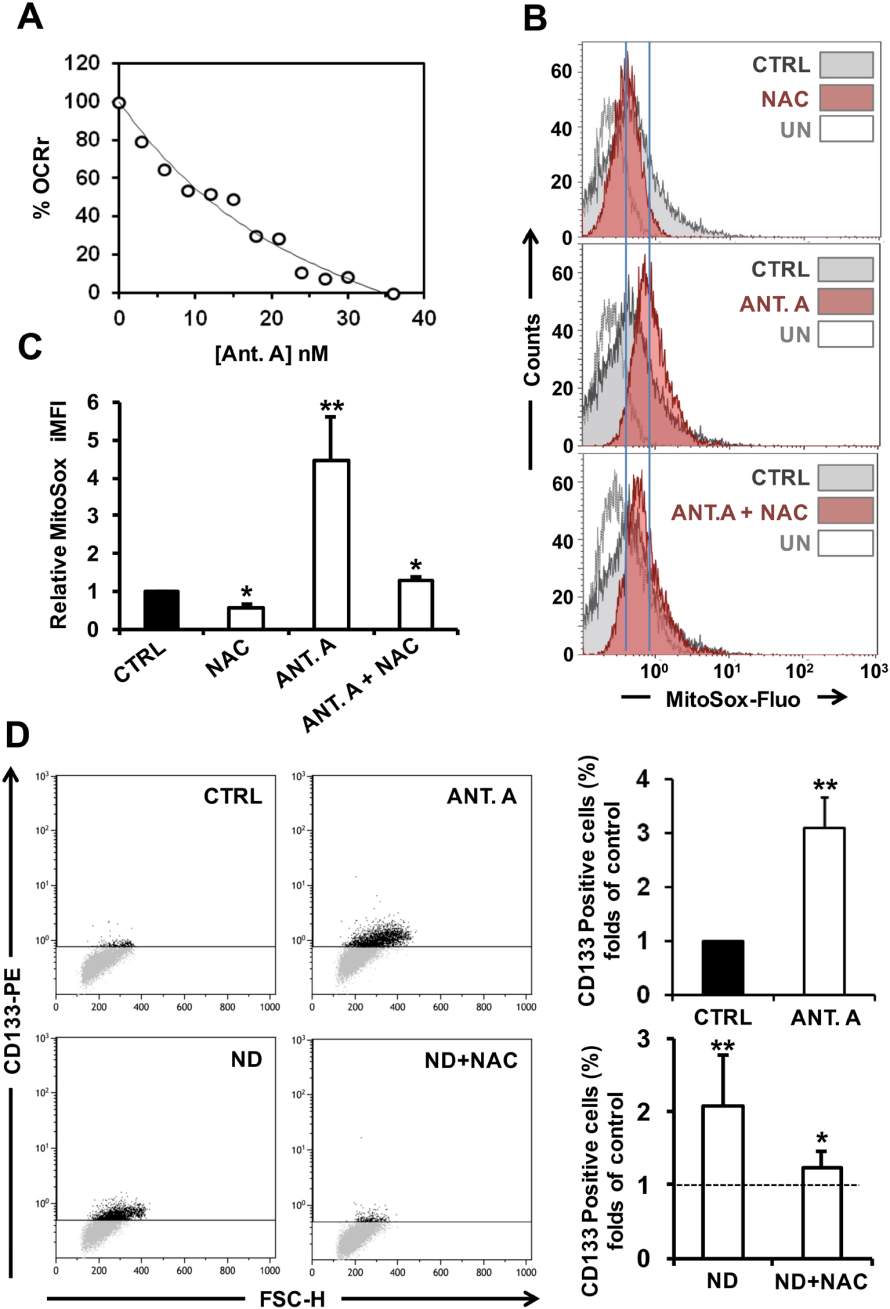
Antimycin A treatment elevates ROS production and induces an increase of CD133 positive HepG2 cells. (**A)** Dose-dependence effect of antimycin A (Ant. A) on cell endogenous respiratory activities. HepG2 cells were exposed to the indicated concentrations of Ant. A and the relative oxygen consumption rate (OCR) was determined. OCR is expressed as the percentage (%) of untreated cells. (**B)** Superoxide anion production was evaluated through flow-cytometry assessment of MitoSox Red fluorescence. HepG2 cells incubated with 10 nM Ant. A for 48 hours presented elevated levels of superoxide production (middle panel), reduced by treatment with 10 mM of the ROS scavenger NAC added 4 h before the analysis (upper and lower panel); unstained controls (UN) are also shown. The bar histogram (**C**) shows the mean intensity of the MitoSox-related integrated fluorescence (iMFI) expressed as fold-change of the untreated cells and are means ± SEM of three independent experiments. ^*^P < 0.05; ^**^P < 0.01. (**D)** Representative dot-plots of flow cytometric analysis of cancer stem cell surface marker CD133 expression. Cells were incubated for 48 h with 10 nM Ant. A and 80 μM Nandrolone ± 10 mM of the ROS scavenger NAC added 6 h before the analysis. The bar histogram on the right shows the percentage of CD133 positive cells expressed as fold-change of the untreated cells (indicated by dashed line) and are means ± SEM of three independent experiments. ^*^P < 0.05; ^**^P < 0.01 vs untreated.

To understand if inhibition of the mitochondrial respiration was *per se* sufficient to induce the observed ND-mediated change of the HepG2 cells immunophenotype we tested the effect of the mitochondrial Cx III inhibitor, antimycin A, at a sub-cytotoxic concentration mimicking the OCR inhibition of ND. Figure 7A shows that 10 nM antimycin A resulted in about 50% inhibition of OCR in HepG2 and in a NAC-sensitive four-fold increase of the basal superoxide production (Fig. 7B,C). This is not surprising given the well documented ROS-generating effect of antimycin on isolated respiring mitochondria as well as in intact cells. Impressively, 10 nM antimycin A caused an increase of the CD133 positive cells comparable with that attained following ND treatment (Fig. 7D). In order to dissect the inhibition of the mitochondrial respiration from the production of ROS, HepG2 cells were treated with nandrolone in the presence of the antioxidant NAC. The result attained clearly shows that NAC was able to fully prevent the enhanced expression of CD133 in nandrolone-treated cells (Fig. 7D).

### Nandrolone affects the differentiation capacity of stem/progenitor cells

**Figure 8:**
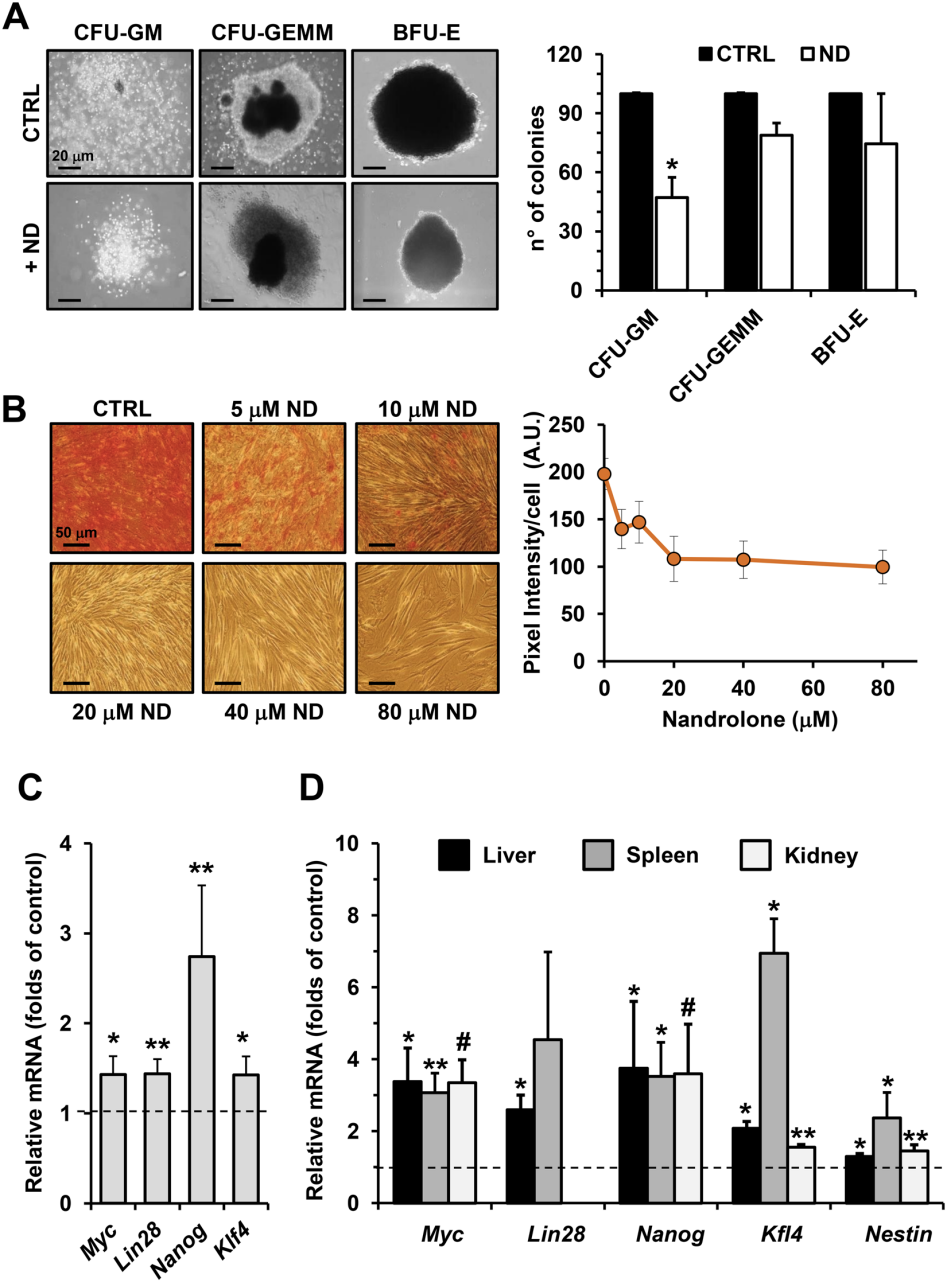
Nandrolone triggers the gain of stemness in several primary non-cancerous cells model. (**A)** Effect of nandrolone on colony formation ability of CD34^+^ HS/PC *in vitro*. Left panel: representative images of CFU-GEMM, CFU-GM and BFU-E scored under an inverted microscope (magnification 40X) are shown. Right panel: quantitative analysis of CFU-GEMM, CFU-GM and BFU-E derived by CD34^+^ cells isolated form human umbelical cord blood with or without 80 μM nandrolone. ND was added to methylcellulose based medium at time of plating and colonies were detected and enumerated after 14 days of culture as described in Material and Methods. Results shown represent the mean of ± SD of two independent experiment, ^*^P < 0.05 vs CTRL. (**B)** Effect of nandrolone on calcific deposition during osteogenic differentiation of dental pulp mesenchymal stem cell (DPSCs). Mineral matrix deposition was assayed by Alizarin Red (red staining) in DPSCs incubated with vehicle (CTRL) cells and cells treated with several doses of nandrolone after 21 days in osteogenic conditions. The graph on the right shows quantitative analysis of alizarin red staining carried out by a densitometric analysis (Image J software). (**C)** Nandrolone treatment induces stemness genes expression during osteogenic differentiation of dental pulp stem cell. Quantitative reverse transcriptase PCR analysis for *Myc, Lin28, Nanog, Kfl4* in DPSCs incubated for 21 days in osteogenic medium with 80 μM nandrolone. Data are the mean ± SEM of normalized transcript levels of five independent experiment from 5 independent donors. Per each gene evaluated, fold change value of transcript level in nandrolone treated cells compared to untreated cells (indicated by dashed line) is shown, ^*^P < 0.05; ^**^P < 0.01. (**D)** Nandrolone elevates stem cells genes expression *in vivo*. Real time-PCR evaluation of stemness gene expression after nandrolone administration in mice model as describe in Material Methods. For each gene, relative expression levels of transcripts of nandrolone treated mice compared to animals treated with vehicle (dashed line) are shown. The graphs show normalization with the reference gene of *Myc, Lin28, Nanog, Kfl4, Nestin* expression in several tissues, as spleen, kidney and liver. Data are the mean ± SEM of normalized transcript levels of 3 independent biological experiment. ^*^P < 0.05; ^**^P < 0.01; ^#^P < 0.001.

Next, we pondered whether the observed effects of ND were cell-type restricted or general. Therefore, we used additional cell models such as the hematopoietic umbilical cord blood (UCB) CD34^+^ stem/progenitor cells and the dental pulp mesenchymal stem (DPSCs) cells, both primary normal stem cells. Using the colony formation unit assay, as a test to assess the proliferative capacity of progenitors in the UCB CD34^+^ cell population, we found that ND-treatment resulted in a 50% significant reduction in the number of granulocyte/monocyte colonies (CFU-GM) as well as a reduction of the common myeloid progenitor-derived colonies (CFU-GEMM) and of the erythroid burst-forming units (BFU-E) (Fig. 8A).

In addition, DPSCs cells, cultured under osteogenic conditions, upon ND treatment displayed a reduced differentiation (mineralization) capacity in a dose dependent manner as shown by microscopy imaging of the alizarin red stained cells (Fig. 8B). Finally, as observed in the hepatoma cell line, also in DPSCs cells, all the stem cells markers resulted to be upregulated (particularly *Nanog*) following ND-treatment (Fig. 8C). These results confirmed the capacity of ND to inhibit cell proliferation and differentiation while fostering towards a more quiescent stem cell-like phenotype.

### Nandrolone triggers *in vivo* tissular gain of stemness

Finally, we validated *in vivo* the effects of nandrolone observed *in vitro*. Mice were treated with intramuscular injections of nandrolone (5 mg/kg twice a week for 6 weeks) or vehicle. One week after the last injection liver, spleen and kidney tissues were collected and subsequently subjected to real-time PCR analysis. As shown in Fig. 8D, also *in vivo* an overall increase in the expression of stemness marker genes was observed in liver, spleen and kidney. This result is remarkable considering that the cellular composition of the sample analysed was largely constituted by differentiated cells.

## Discussion

Nandrolone is a testosterone derivative, known as one of the most commonly used androgens and anabolic steroids (AAS) to improve athletes physical performance exhibiting strong anabolic effects and weak androgenic effects. In addition, nandrolone has found wide clinically application including oncological treatment with therapeutic outcomes depending on cancer type^30–32^. In this context, the first aim of this study was to investigate the effects of nandrolone in hepatocellular carcinoma, where androgens involvement has been already described, by using the hepatoma-derived cell line HepG2. Moreover, extension of the obtained observations to normal cell types and in *in vivo* model allowed us to better clarify the impact of chronic exposure to AAS in healthy people.

First, we looked at the cell growth rate that appeared clearly slowed upon nandrolone treatment while cell viability was not significantly affected. Consistent with this observation, the cell cycle analysis showed a lower percentage of cells in S phase associated to a higher percentage in G2 phase of treated cells compared to the control, thus suggesting a G_2_/M cell cycle arrest. Furthermore, the expression of positive and negative regulators of cell proliferation such as Cyclin D1, Cyclin E, Cdk1/2 and p21, p53 resulted to be down- and up-regulated respectively, further confirming a strong repression of cell proliferation induced by nandrolone in HepG2 cells. An inhibitory effect on cell growth exerted by nandrolone has been already reported on other cell lines such as the Leydig cells^2,33^.

Slowing of cell growth is generally linked to modification of the cell metabolism because of the lower energy needs; this might be a consequence, adapting the cellular bioenergetic to the more quiescent cellular state, or it may cause *per se* a dampening of cell proliferation. To deepen this aspect, we evaluated the bioenergetic metabolic fluxes in intact cells by Seahorse methodology. As expected, the mitochondrial oxygen consumption rate appeared to be lower in cells treated with nandrolone while the glycolytic flux was unaffected. Consistent with this observation, measurements of the respiratory chain complexes activities revealed a strong inhibition of complexes I and III whereas the complexes II and IV activities were not significantly affected. Since the amount of specific subunits of all the respiratory complexes resulted unchanged following nandrolone-treatment this suggested a direct effect exerted by the compound on the mitochondrial respiration rather than on the biogenesis of the respiratory complexes.

This was confirmed by assessing the direct effect of nandrolone on the enzymatic parameters of CxIII which resulted in a competitive inhibition with the co-substrate ubiquinol. Consistent with this observation it has been reported that administration of ND in emphysematous hamsters decreased the activity of succinate:cytochrome c oxidoreductase compared with ND treatment in normal hamsters^34^. In addition to compromise the mitochondrial respiratory chain activity, inhibition of CxIII is well-known to enhance ROS production^35^. This appears to be due to accumulation of reactive radical intermediates, which are intrinsic to the catalytic cycle of the CxIII, and in addition to a phenomenon defined as “reverse electron transfer” that move reducing equivalents from ubiquinol back through the CxI, which becomes under this condition a further ROS generator^28,36^. Accordingly, we detected enhanced production of ROS, following nandrolone-treatment, traceable back to the mitochondrial compartment.

Depression of mitochondrial oxygen consumption in cancer cells is associated to a stem cell-like phenotype due to the lower energy demand characterizing the metabolic quiescent cancer stem cells^37,38^. Indeed, mitochondrial OxPhos is mandatory to engage epithelial/mesenchymal transition^39^. Therefore, we decided to analyze the gene expression profile of the stemness markers *Lin28, Myc, Klf4* and *Nanog* that resulted significantly upregulated in HepG2 cells following nandrolone treatment. This result is consistent with a previous report showing that the androgen/androgen receptor axis promotes cancer cell stemness by activation of *Nanog* transcription^21^. In this study the authors found that treatment of hepatocellular carcinoma cell lines with dihydrotestosterone (DHT) resulted in increased expression of the *Nanog* gene with a fold change that was comparable with attained in our study following ND treatment of HepG2 cells. In addition, the number of CD133^+^ cells, identified as more immature cells, expanded upon nandrolone treatment suggesting that the compound causes a shift toward a stemness phenotype. Notably, the CxIII inhibitor antimycin A mimicked the effect caused by nandrolone up-regulating the CD133^+^ expression. Insightfully, the ROS scavenger NAC prevented the enhancement of the nandrolone-mediated number of CD133^+^ cells pointing to an important role played in this context by redox signaling.

Dysfunctions of the mitochondrial respiratory chain have been reported both to inhibit and to promote cancer depending on the specific respiratory complex involved^40,41^. In particular metformin, a first line drug for treatment of type 2 diabetes, is thought to exert its action by inhibiting the mitochondrial complex I^42^. Notably, epidemiological studies indicate that metformin protects against cancer development^43^. In the context of our study this would suggest that the observed inhibition of complex III is more important than that of complex I for the stemness induction in cancer cells.

Further studies are, however, needed to clarify whether the nandrolone-induced metabolic shift and/or redox signaling induce de-differentiation of the cells or the modified metabolic environment favors the selective proliferation of pre-existing cancer stem-like cells^44^. An emerging concept in oncology is that a dynamic interconversion among different subsets of CSCs, and between CSC and non-CSCs continually occurs during the development of a tumor^45^. Altered conditions could trigger the gain of stemness, some of them including: EMT-MET, epigenetic modifications, microenvironment influence and selective stimuli such as altered life style and chemotherapy^20^. The above described unexpected finding led us to explore the effect of nandrolone in normal stem cells also considering that nandrolone is chronically administered in healthy young people. Interestingly, nandrolone exerted both in cord blood-derived human hematopoietic CD34^+^ cells and in mesenchymal dental pulp stem cells a negative effect on cell differentiation evaluated by colony formation and osteoblastic lineage differentiation ability respectively. Moreover, also mesenchymal pulp stem cells showed up-regulation of all the stemness markers tested upon nandrolone-treatment, indicating a general effect of the drug regulating the stemness phenotype also in normal primary stem/progenitor cells. Finally, up-regulation of the stemness markers was also found *in vivo*, in healthy mice, particularly in kidney, liver and spleen, with a stronger upregulation in liver and spleen, likely because they are tissues richer in stem cells.

A survey of the literature on the effect of nandrolone on cell differentiation reports conflicting results. Indeed, if nandrolone negatively affects neural stem cell proliferation and neurogenesis in rat brains^46^ on the other hand it appears to modulate proliferation of myoblasts^47,33^, to increase satellite cell number^48^, to promote erythropoiesis^49,50^, to foster osteoblasts proliferation and differentiation^51^. In particular, it has been reported that the nandrolone-induced proliferation of different cancer cell lines is linked to the activation of the insulin-like growth factor 1 receptor (IGFR1)-mediated signaling^52,53^ with the maximal effect attained at a concentration as low as 1 μM of the drug. Likely, the sub-pharmacological dosage of the drug used in most of the above-mentioned studies elicits responses which are overwhelmed by those evocated by the concentration of nandrolone used in this study. Indeed, treatment of HepG2 with ND under our experimental conditions did not result in any change neither of the IGFR1 expression or IGF-1 release or Akt-signaling activation (data not shown). The choice to test a concentration of 80 μM nandrolone throughout this study was dictated, in addition to the absence of adverse effects in terms of cytotoxicity on *in vitro* cell culture, by the fact that it would roughly correspond to the administration of about 200 mg of nandrolone decanoate, which is the suggested anabolic dosage for sportsman and bodybuilders but about 10 times higher than that prescribed for therapeutic purposes.

**Figure 9:**
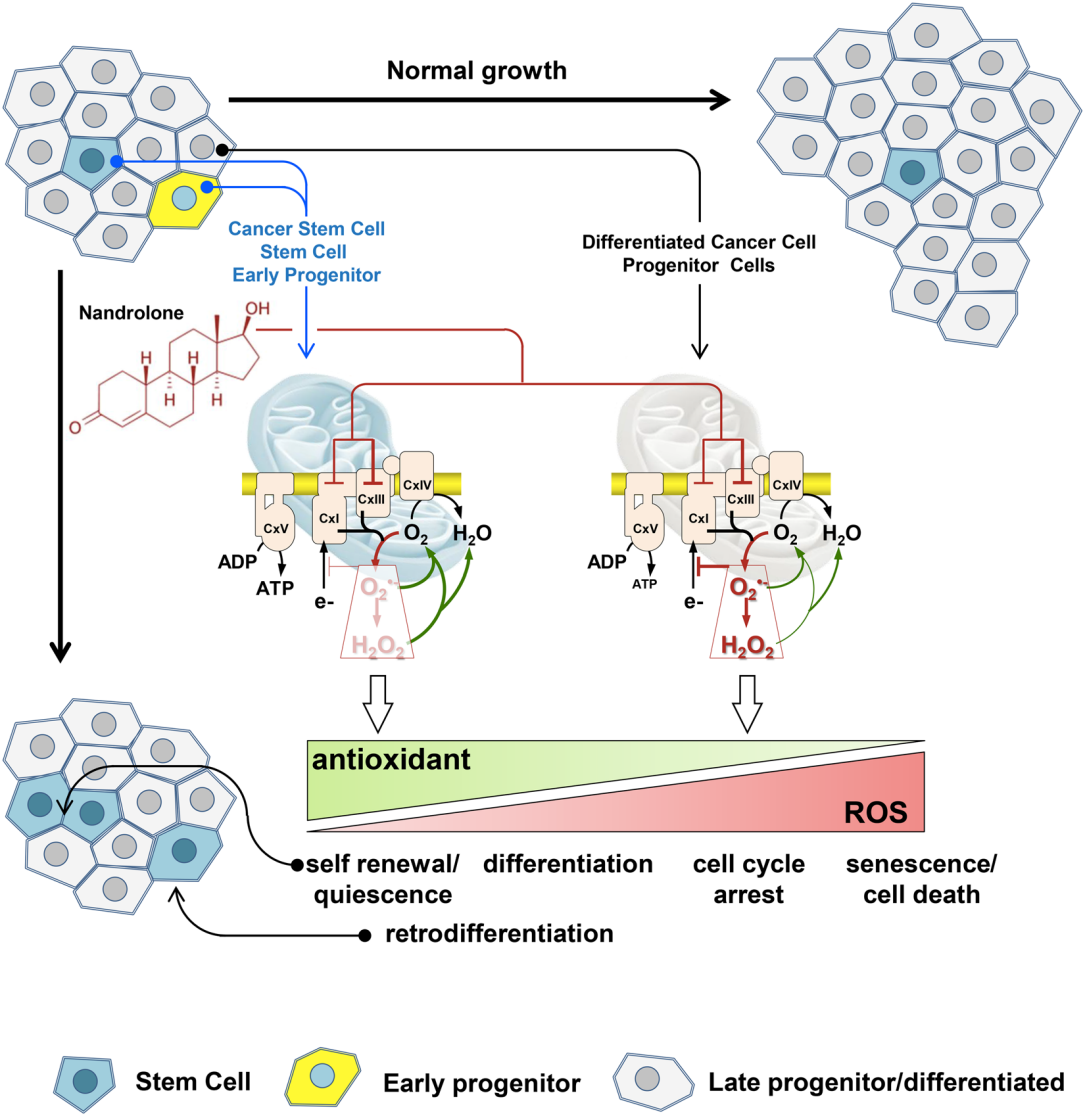
Proposed mechanism of the differential action of nandrolone on normal/cancer stem and differentiated cells. The hypothesis is put forward that by inhibiting the mitochondrial respiratory chain CxIII, nandrolone induces a pro-oxidative setting (red arrowed lines) that depending on the cellular antioxidant supply (green arrowed lines) establishes a differential redox signalling. A low increase of reactive oxidant species would favour self-renewal/quiescence of normal or cancer stem cells and possibly retro-differentiation of early progenitors, all equipped with a robust antioxidant armoury. Conversely, a higher pro-oxidative state, such that caused by nandrolone in late progenitor or differentiated cells will induce cell cycle arrest and possibly cell death. See Discussion for further details.

Collectively, our results show, for the first time, that nandrolone induces mitochondrial dysfunction and dampening of cell growth in hepatoma cells contributing to the maintenance/expansion of a stem cell-like phenotype. We hypothesize that this simultaneous and paradoxical effect relies on a different cell-type response to the compound whereby differentiated cancer cells or mature progenitors reduce their growth capability whereas cancer stem cells or early progenitors enhance their properties (Fig. 9).

On the basis of the here-reported results we are tempted to indicate in a differential ROS-mediated signaling the distinct cell phenotype-dependent response to the drug. It is well-established that reactive oxygen (and nitrogen) species when kept below the harmful condition defined as oxidative stress function as messengers eliciting a number of physiological responses^54–56^. These encompass control of stem cell self-renewal and maintenance of quiescence, proliferation of progenitors, activation or pre-conditioning for differentiation, block of cell cycling, senescence and cell death. In addition, also de-differentiation appears to be conditioned by the cellular redox tone. The specific response appears to depend on the level of the reactive species, their intracellular compartmentalization and chemical properties. The amount of generated reactive species is kept under control by the armory of cellular chemical and enzymatic antioxidants. Normal and cancer stem cells proved to express higher levels of antioxidant enzymes as compared with their committed counterparts^57–59^. Accordingly to this notion it is conceivable that a pro-oxidative condition might elicit different and even opposite responses depending on the antioxidant cellular phenotype. Thus, the apparent beneficial antitumoral effect on differentiated cancer cells is counterbalanced by the harmful enrichment of the cancer stem cells compartment, which appears to be the major determinant of tumorigenesis^60,61^.

Most notably, nandrolone pushes toward a stemness phenotype also different kind of healthy stem cells and healthy tissues. This represents the main alarming factor because the exposure to AAS starts at an earlier age; in fact, a significant increase of their use among adolescent athletes and specific subsets of the general population (high school and college students) has been observed^62,63^ and the non-medical use of AAS is considered a major and widespread public health issue^30^. Socas *et al*.^9^ reported two very different cases of adult male bodybuilders who developed hepatocellular adenomas following AAS abuse. In both cases the patients have evolved favorably and the tumors tended to regress after the withdrawal of AAS. Recently, Shalaby AM *et al*.^64^ described liver alterations detected in nandrolone-treated rats (i.e. inflammatory cellular infiltration, severe vacuolar cytoplasmic degeneration, apoptotic hyperchromatic nuclei and partial loss of mitochondrial cristae in the hepatocytes) rescued by nandrolone withdrawal.

It has been suggested that the incidence of cancer in different tissue is strictly positively correlated to the number of stem cell divisions in the lifetime occurring in them^65^. On this basis, it can be hypothesized that the chronic administration of nandrolone, favoring the persistence and viability of stem cells in different tissues, could represent a preconditioning that, in addition to multiple hits, could enhance the risk of carcinogenesis onset especially in stem cell-rich tissues such as liver.

## Materials and Methods

### Cell cultures and treatment

Human hepatoma-derived cell line (HepG2) from the European Collection of Cell Cultures (ECACC Salisbury, UK) were maintained at 37 °C (5% CO_2_) in DMEM supplemented with 10 mM Hepes, 10% inactivated FBS, 2 mM glutamine, 100 U/ml of penicillin and 100 μg/ml of streptomycin. For *in vitro* experiments, cells at approximately 50% confluence were treated for 72 h with 80 µM nandrolone (Vetranal-analytical standard, Sigma–Aldrich, St. Louis, MO). The mitochondrial complex III inhibitor antimycin A (Sigma–Aldrich, St. Louis, MO), when indicated, was added to the HepG2 culture at 10 nM for 72 h. Untreated cells, used as control, were supplemented with vehicle only (ethanol) that never exceeded 0,002% (V/V). For antioxidant treatment, cells were incubated for 72 h with 80 µM nandrolone ± 10 mM N-Acetyl Cysteine (NAC) added 4 h before the analysis. The cellular morphology was observed at inverted optical microscope (Axio Vert A1, Zeiss, Oberkochen, Germany). 3D culturing of HepG2 cells was performed as previously described^66^. To evaluate the effect of nandrolone to spheroid formation, 80 μM nandrolone was added to cell suspension when seeded into ultra-low attachment plates and the culture was maintained for 3 days or 7 days. Spheroids were photographed on an inverted optical microscope and their diameter was measured using the ZEISS ZEN imaging software. Human Dental pulp mesenchymal stem cells (hDPMSc) were cultured and differentiated in the osteoblast lineage as described elsewhere;^67^ the capacity of differentiated hDPMSc to produce calcium-rich deposits was analyzed by using alizarin red staining (ARS) as previously described^67^.

### Mice model animals and animal care

The care and treatment of all animals were carried out in accordance with the EU Council Directive 86/609/EEC, the Animals Scientific Procedures Act 1986 and approved by the Faculty of Medicine and Surgery Animal Care and Use Committee, University of Malta. Mice were treated with intramuscular injections (IM) of ND 5 mg/kg or peanut oil twice a week in the hind-limb for 6 weeks according to the literature^68^. One week after the last injection, mice were killed via cervical dislocation. Spleen, kidney, liver tissues were collected and immediately frozen in liquid nitrogen, stored at −80 °C and subsequently subjected to real-time PCR analysis as described later.

### Cell proliferation, viability, cell cycle and apoptosis analysis

Cell growth curves of HepG2 cells were obtained as previously described^69^. Cell viability was measured by the MTS assay using the tetrazolium compound (3-(4,5-dimethylthiazol-2-yl)-5-(3-carboxymethoxyphenyl)-2-(4-sulfophenyl)-2H-tetrazolium), inner salt (CellTiter 96 AQueous MTS Reagent Powder, Promega) and the electron coupling reagent, phenazine methosulfate, PMS (Sigma Aldrich, Saint Louis, MO, USA) in 3 × 10^3^ cells seeded in a 96-well plate. The absorbance of the formazan at 490 nm was measured directly using the Plate Reader (Das srl, Italy) with a reference filter at 630 nm. All measurements were performed in triplicate for each assay. Absorbance of untreated cells (basal) was considered 100%.

For cell cycle determination, cells treated for 72 h with 80 µM nandrolone were harvested into single-cell suspensions, collected by centrifugation and washed twice with PBS. The cells were suspended and fixed with 1 ml of 70% cold ethanol, added dropwise with gentle vortex and then incubated at 4 °C overnight at −20 °C. After further washing, 200 μl of ethanol-fixed cells were incubated with the propidium iodide (PI) staining solution (100 µg/mL RNAse A, 40 µg/mL propidium iodide, Sigma) for 30 minutes at room temperature. A total of 10^4^ events was acquired and analyzed by flow cytometry system (Navios, Beckman Coulter, Brea, CA, USA) for cell cycle analysis. Cell cycle distributions were analyzed in the form of percentage of cells in the G1, S and G2 phases by Kaluza Analysis 1.3 software (Beckman Coulter). Apoptosis was detected by flow cytometry (Navios, Beckman Coulter, Brea, CA, USA) following staining of cells for Annexin-V-FITC and PI (BD Biosciences), after 72 h of incubation with nandrolone (80 μM). A total of 10^4^ events for each sample were acquired.

### Measurement of metabolic flux analysis, lactate and mitochondrial respiratory complex enzymatic activity

Oxygen consumption rate (OCR) and extra-cellular acidification rate (ECAR) were measured with a XFe96 Extracellular Flux Analyzer (Seahorse Bioscience, Billerica, MA, USA) as previously described by Scrima R^70^ in low-buffered DMEM supplemented with 1 mM pyruvate, 2 mM glutamine, 10 mM glucose for OCR measurements or with 1 mM pyruvate, 2 mM glutamine for ECAR measurements. Briefly, for OCR analysis, after measuring basal respiration, oligomycin (1 μM), FCCP (1 μM), and rotenone + antimycin A (1 μM + 1 μM) were injected into each well sequentially to assess coupling of respiratory chain, maximal and non-mitochondrial oxygen consumption, respectively. For ECAR analysis, glycolytic flux (basal glycolysis, glycolytic capacity, and glycolytic reserve) was analyzed by the sequential addition of 10 mM glucose, 1 μM oligomycin, and 100 mM 2-deoxyglucose. OCR and ECAR values were normalized to protein content in each well, determined by BCA assay (Thermo Scientific, Dreieich, Germany). Lactate measurement was performed by a lactate colorimetric assay kit (Abcam, Cambridge, MA, USA) following the manufacturer’s protocol and normalized to cell number. Measurement of the specific activity of mitochondrial NADH:ubiquinone oxidoreductase (complex I), succinate:ubiquinol oxidoreductase (complex II), ubiquinone:cytochrome c oxidoreductase (complex III), and cytochrome c oxidase (complex IV) was carried out spectrophotometrically on frozen–thawn and ultrasound-treated cells as previously described^71,72^.

### Clonogenic assay

Clonogenic assay was performed as previously described^73^. Briefly, isolated human umbilical cord blood (hUCB) CD34^+^ cells (2 × 10^5^ cells/ml) were resuspended in RPMI 1640 medium supplemented with 2% FBS, and then mixed with methylcellulose base cultures (MethoCult-H4434; StemCell Technologies, Inc., Vancouver, BC, Canada) with or without 80 µM nandrolone. Two weeks later, colony‐forming unit‐granulocyte/macrophage (CFU‐GM) colonies, burst‐forming unit‐erythroid (BFU‐E) colonies and colony- forming unit-granulocyte, erythroid, macrophage megakaryocytes (CFU-GEMM) colonies were scored under an inverted microscope (magnification 40X). Each assay was performed in duplicate.

### Western blotting analysis

Immunoblots were performed as previously described^69^. Briefly, membranes were probed with the following primary antibodies: Cyclin D1 (1:1000; Cell Signaling Technology), Cyclin E (1:1000, EDM Millipore Temecula, CA, USA), Cdk1/2 (1:1000, Santa Cruz Biotechnology, Santa Cruz, CA, USA), p53 (1:1000 Cell Signaling Technology, Danvers, MA, USA), p21/WAF1/Cip1 clone CP74 (1:1000, EDM Millipore Temecula, CA, USA), MitoProfile Total OXPHOS Human WB Antibody cocktail (1:500; Abcam Cambridge, UK) and β-Actin (1:5000; Sigma Aldrich, St. Louis, MO, USA). The secondary antibody was horseradish peroxidase-conjugated (1:2500; Cell Signaling Technology) and the signals developed by enhanced chemiluminescence kit (Clarity Western ECL Substrate, Bio-Rad), acquired by ChemiDoc Imaging System XRS + (BioRad) and analyzed for densitometry with the ImageJ Lab 4.1 software.

### RNA extraction, reverse transcription and real‐time‐polymerase chain reaction analysis

Total cellular RNA was isolated from cell cultures and mouse homogenized sample tissues using TRIzol REAGENT (Invitrogen) according to the manufacturer’s instructions. Quantitative real‐time polymerase chain reaction (PCR) assays were performed in duplicate as described elsewhere^74^ using the SYBR Green QuantiTect Primers purchased from QIAGEN (Basel, Switzerland) and listed in Supplementary Table 1. Reactions were set up in 96-well plates using a LightCycler 480 real‐time PCR Instrument (Roche Diagnostics). The relative amounts of target genes were normalized to GAPDH or RN18S expression by Light Cycler 480 Software version 1.5 (Roche Diagnostics) using the 2^−ΔΔCt^ method.

### Flow cytometric detection of ROS

To measure intracellular and mitochondrial ROS, we used 4 μM 2,7-dichlorofluorescin diacetate (DCF-DA) for cellular peroxide detection or 5 μM MitoSOX for superoxide specifically produced by mitochondria, respectively. Both probes, purchased from Molecular Probes (Eugene, OR, USA) were added to cell suspension and incubated, protected from light, at 37 °C for 15 min. Stained samples were acquired using Navios flow cytometer and analyzed by Kaluza Analysis 1.3 software (Beckman Coulter, Indianapolis, USA).

### Live cell imaging of ROS

Cells cultured at low density on fibronectin-coated 35-mm glass-bottom dishes (ibidi GmBH, Gräfelfing, Germany) were incubated for 20 minutes at 37 °C with 4 μM DCF-DA (2,7-dichlorofluorescin diacetate) or 5 μM MitoSox (Molecular Probes, Eugene, OR). Stained cells were washed with PBS and examined by a Leica TCS SP8 confocal laser scanning microscopy system (images collected using a 60X objective [1.4 NA]). Acquisition, storage, and analysis of data were performed with LasX software from Leica or ImageJ 1.48 (Wayne Rasband, NIH, USA, http://imagej.nih.gov/ij).

### Cytofluorimetric analysis of CD133 expression

Flow cytometric detection of surface marker CD133 in HepG2 cells was performed as previously described^75^. Briefly, cell suspensions (0.6 × 10^6^ cells/ml per condition) were stained with mouse anti-human CD133/2 antibody conjugated to R-Phycoerythrin (PE) (1:10, Miltenyi Biotec) in the dark at 4 °C for 15 min in a PBS/EDTA buffer containing 2 mM EDTA and 0,5% BSA. After washing, cells were resuspended in PBS and a total of 10^4^ events for each sample was acquired and analyzed by Navios flow cytometer with Kaluza Analysis 1.3 software (Beckman Coulter, Indianapolis, USA).

### Statistical analysis

Experimental data are reported as mean ± standard deviation or mean ± standard error of the mean. Data were compared by an unpaired Student’s-*t*-test or, when necessary, by 2-way ANOVA followed by a post-hoc Bonferroni test. Differences were considered statistically significant when the P < 0.05. All analyses were performed using Graph Pad Prism (Graph Pad software, San Diego, CA, USA).

## Supplementary information

Supplementary Information.
