# Supplementary material for: Nandrolone induces a stem cell-like phenotype in human hepatocarcinoma-derived cell line inhibiting mitochondrial respiratory activity
Source: Sci Rep. 2020 Feb 10;10:2287. doi: 10.1038/s41598-020-58871-1 (PMC7010785; doi:10.1038/s41598-020-58871-1)
Supplement: Supplementary file 1 — Supplementary Information. [file 41598_2020_58871_MOESM1_ESM.pdf]

Supplementary Information

Nandrolone induces a stem cell-like phenotype in human hepatocarcinoma-derived cell line inhibiting mitochondrial respiratory activity

Francesca Agriesti [1], Tiziana Tataranni [1], Consiglia Pacelli [2], Rosella Scrima [2], Ilaria Laurenzana [1], Vitalba Ruggieri [1], Olga Cela [2], Carmela Mazzoccoli [1], Monica Salerno [3], Francesco Sessa [2], Gabriele Sani [4,5,6], Cristoforo Pomara [3], Nazzareno Capitanio [2], Claudia Piccoli *[1,2]

[1] Laboratory of Pre-Clinical and Translational Research, IRCCS-CROB, Referral Cancer Center of Basilicata, 85028 Rionero in Vulture, Italy. [2] Department of Clinical and Experimental Medicine, University of Foggia, via L. Pinto c/o OO.RR., 71100 Foggia, Italy. [3] Department of Medical, Surgical Sciences and Advanced Technologies "G.F. Ingrassia", University of Catania – A.O.U. "Policlinico - V. Emanuele" (via S. Sofia, 87 – Sector 10, Building B – 95123) Catania, Italy. [4] NESMOS Department (Neurosciences, Mental Health, and Sensory Organs), Sapienza University of Rome, Italy. [5] School of Medicine and Psychology, Sant'Andrea Hospital, Rome, Italy. [6] Centro Lucio Bini, Rome, Italy.

Francesca Agriesti ORCID: https://orcid.org/0000-0001-8911-7691

***Corresponding author**:
Claudia Piccoli, Department of Clinical and Experimental Medicine,
University of Foggia,
via L. Pinto c/o OO.RR., 71100 Foggia, Italy.
e-mail: claudia.piccoli@unifg.it
Phone number: +39 0881 588060
Fax number: +39 0972 723509
ORCID: https://orcid.org/0000-0001-9739-4680

Supplementary Figure S1. Full-length blots of Figure 2B

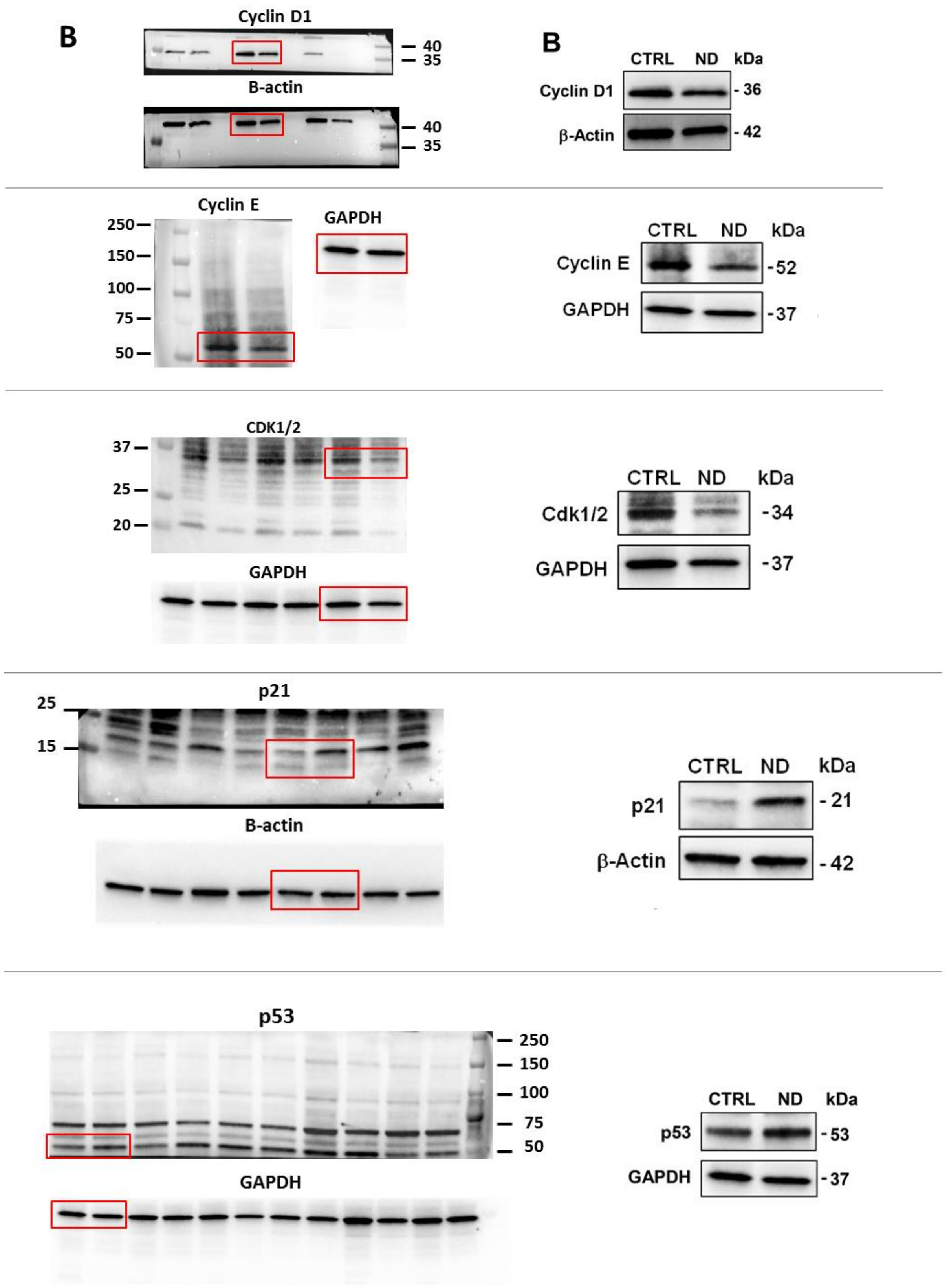

Supplementary Figure S2. Effect of nandrolone treatment on lactate production in HepG2 cells. The bars are average ± SEM of three independent experiment. See Materials and Methods for details.

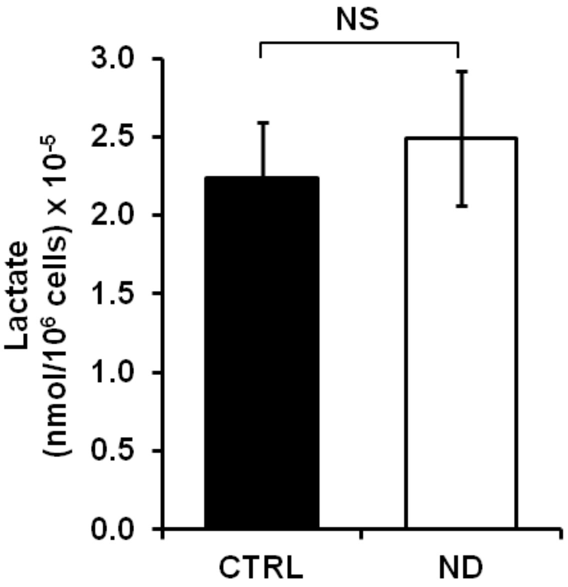

Supplementary Figure S3. Full-length blots of Figure 4B

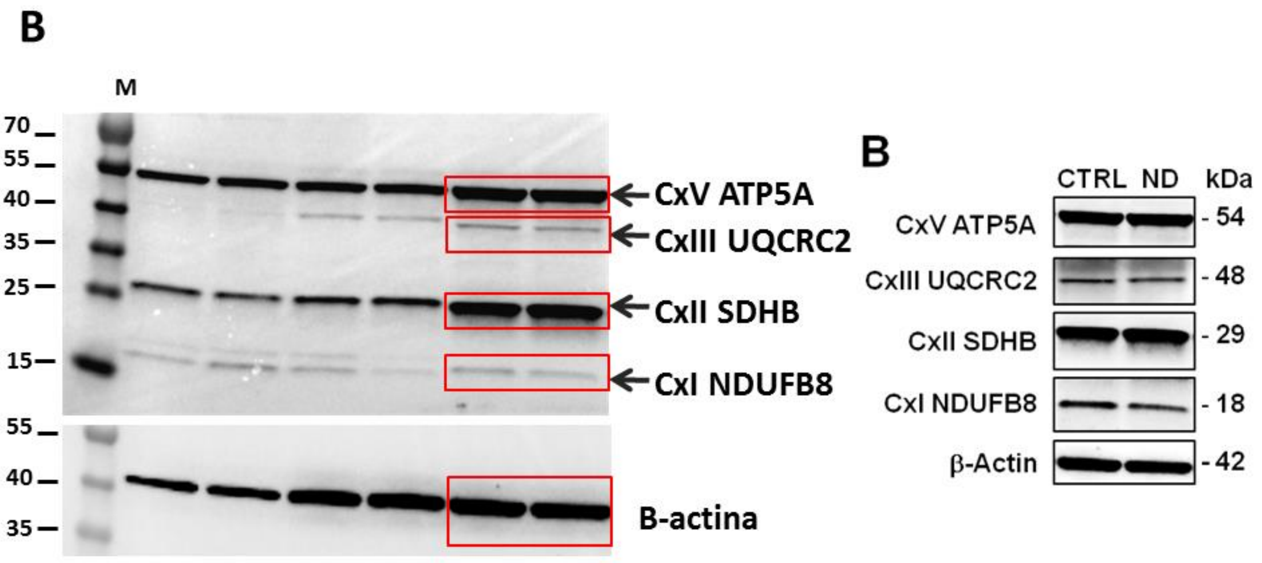

Supplementary Figure S4. Evaluation of Nandrolone effect on the kinetic parameters of Ubiquinone:cytochrome c oxidoreductase (CxIII). Left graph: Lineweaver-Burk (L/B) plot illustrating inhibition of CxIII inhibition tested (in absence or) in the presence of different concentrations of ND as follows: 160 μM (○), 80 μM (▲), 40 μM (■). Graph on the right: secondary plot of slope against [ND] to determine the Ki (300 μM). The inhibition is competitive and the slopes were calculated from the L/B plot. CxIII activity was assayed in mitochondria isolated from beef heart by differential centrifugation as previously described [Srima R. et al. Plos One 12, e0188683 (2017)]. Mitochondria (1.0 mg prot/ml) were suspended in 0.25 M sucrose, 5 mM Hepes, 1 mM EDTA, 5 mM $MgCl_2$ and the kinetic parameters of CxIII were assessed spectrophotometrically (in the presence of 1.0 μg/ml rotenone *plus* 2 mM KCN) by following the initial rate of 50 μM ferri-cytochrome *c* reduction (ε550nm = 21.1 mM^{-1} cm^{-1}) elicited by graded concentrations of $dUQH_2$ (20, 50, 100, 200, 300 μM) as electron donor in the absence or presence of graded concentrations of nandrolone (40, 80, 160, μM). The rates were corrected for the residual cytochrome c reduction in the presence of 1 μg/ml antymicin A. The attained data points were plotted as double reciprocal plot and Dixon plot and linearly fitted by least square analysis using EnzFitter (Biosoft).

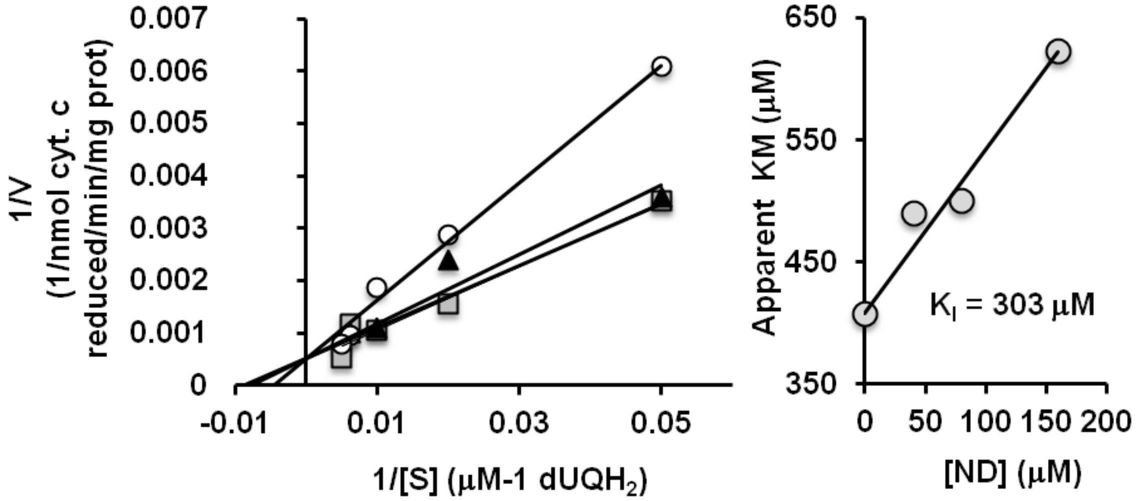

Supplementary Figure S5. Effect of nandrolone treatment (ND) on the expression of the housekeeping gene GAPDH in HepG2 cells and derived spheroids. Bars in the histogram are averages ± SD of at least 6 independent assays under each condition and show the cycle threshold (Ct) of the RT-PCR analysis of the GAPDH expression (see Materials and Methods of the main text and Supplementary Table 1 for details). Black bars show the effect on cultured HepG2 cells of 72 h treatment with vehicle (ethanol) or 80 μM nandrolone. White bars show the effect of nandrolone on forming spheroid with 80 μM of the drug supplemented to the cell suspension at the beginning of the spheroid formation protocol (see Materials and Methods of the main text for details) and assessed after 72 h.

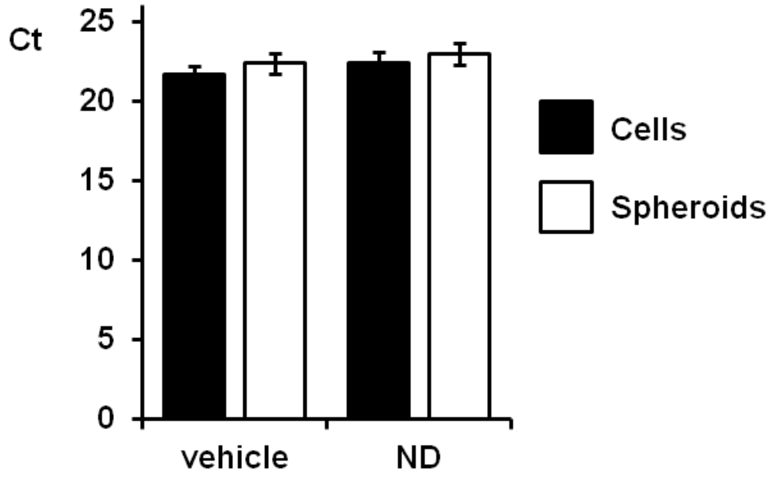

Supplementary Table 1. Features of the primers used for real-time polymerase chain reaction

Gene	Organism	T_{ann} (°C)	Length (bp)	NCBI Ref Seq or sequence
Nanog	Mouse (Mus musculus)	55	114	NM_028016
Nestin	Mouse (Mus musculus)	60	81	NM_016701
Myc	Mouse (Mus musculus)	60	60	NM_001177352
Lin28	Mouse (Mus musculus)	55	116	NM_145833
Sox17	Mouse (Mus musculus)	60	77	NM_011441
Kfl4	Mouse (Mus musculus)	60	66	NM_010637
Rn18S	Mouse (Mus musculus)	60	97	Forward: acaggattgacagattga<br>Reverse: tatcggaattaaccagaca
Nanog	Homo sapiens	55	90	NM_024865
Myc	Homo sapiens	60	129	NM_002467
Lin28	Homo sapiens	55	126	NM_024674
Sox2	Homo sapiens	60	64	NM_003106
Kfl4	Homo sapiens	60	72	NM_004235
Gapdh	Homo sapiens	60	135	Forward: aggctgagaacgggaagc<br>Reverse: ccatggtggtgaagacg
